# Metabolomics Applied to Cord Serum in Preeclampsia Newborns: Implications for Neonatal Outcomes

**DOI:** 10.3389/fped.2022.869381

**Published:** 2022-04-25

**Authors:** Xiaoxu Wang, Jieying Liu, Xiangyi Hui, Yingna Song

**Affiliations:** ^1^Department of Obstetrics and Gynecology, National Clinical Research Centre for Obstetric and Gynecologic Diseases, State Key Laboratory of Complex Severe and Rare Diseases, Peking Union Medical College Hospital, Chinese Academy of Medical Sciences and Peking Union Medical College, Beijing, China; ^2^State Key Laboratory of Complex Severe and Rare Diseases, Medical Research Center, Peking Union Medical College Hospital, Chinese Academy of Medical Sciences and Peking Union Medical College, Beijing, China

**Keywords:** preeclampsia, neonate, SGA, metabolomics, pathway

## Abstract

Preeclampsia (PE) is one of the leading causes of maternal and perinatal morbidity and mortality. However, it is still uncertain how PE affects neonate metabolism. We conducted an untargeted metabolomics analysis of cord blood to explore the metabolic changes in PE neonates. Umbilical cord serum samples from neonates with preeclampsia (*n* = 29) and non-preeclampsia (non-PE) (*n* = 32) pregnancies were analyzed using the UHPLC-QE-MS metabolomic platform. Different metabolites were screened, and pathway analysis was conducted. A subgroup analysis was performed among PE neonates to compare the metabolome between appropriate-for-gestational-age infants (*n* = 21) and small-for-gestational-age (SGA) infants (*n* = 8). A total of 159 different metabolites were detected in PE and non-PE neonates. Creatinine, N4-acetylcytidine, sphingomyelin (D18:1/16:0), pseudouridine, uric acid, and indolelactic acid were the most significant differential metabolites in the cord serum of PE neonates. Differential metabolite levels were elevated in PE neonates and were involved in the following metabolic pathways: glycine, serine, and threonine metabolism; sphingolipid, glyoxylate, and dicarboxylate metabolism; and arginine biosynthesis. In PE neonates, SGA neonates showed increased levels of hexacosanoyl carnitine and decreased abundance of 3-hydroxybutyric acid and 3-sulfinoalanine. Taurine-related metabolism and ketone body-related pathways were mainly affected. Based on the UHPLC-QE-MS metabolomics analysis, we identified the metabolic profiles of PE and SGA neonates. The abundance of metabolites related to certain amino acid, sphingolipid, and energy metabolism increased in the umbilical cord serum of PE neonates.

## Introduction

Preeclampsia (PE) is a major multisystem complication in pregnancy, characterized clinically by new hypertension after 20 weeks of gestation with proteinuria or other clinical signs of impaired end-organ function, including abnormalities of the kidneys, liver, brain, and platelets (1). PE is one of the main causes of iatrogenic premature births (2) and perinatal deaths worldwide, accounting for approximately 10–20% of perinatal mortality (3). It is commonly accepted that the pathophysiology of PE is attributed to the abnormal formation of spiral arteries in the maternal placenta, giving rise to placental oxidative stress and leading to inappropriate and exaggerated maternal responses involving endothelial dysfunction and systemic inflammation (4). However, several studies have shown that preeclampsia has adverse effects on preterm birth and fetal growth restriction in the perinatal period and has long-term effects on offspring in adulthood, manifested as conditions such as an increased risk of hypertension, cardiovascular disease, neurological diseases, and Alzheimer’s disease (5).

Metabolomics, a newly developed subject following genomics, transcriptomics, and proteomics, is an important component of systems biology. Metabolomics refers to the quantitative measurement of metabolic changes caused by pathophysiological changes and the analysis of changes in all endogenous molecule substances at a certain moment to determine the overall state of the organism. Metabolites hold huge potential sources of biomarkers and can also reveal the metabolic process of diseases, assisting in exploring their pathogenesis.

Several studies have demonstrated the metabolome perturbation of PE and discovered characteristic metabolites in the blood, urine, and placenta of pregnant women with PE, including lipids (6), fatty acids (7, 8), metabolites associated with lipid transport (9, 10), amino acids and related metabolites (11, 12), and purine-related metabolites (13). These studies have mainly focused on pregnant women with preeclampsia; however, there are relatively few studies on newborns. A previous study reported an increased level of certain metabolites in the cord blood of PE neonates, including the urea cycle and carnitine synthesis (14). A tryptophan-targeted metabolomics study showed changes in tryptophan metabolites in PE fetal plasma, and 3-hydroxyanthranilic acid was only present in fetal circulation and not in the maternal circulation (15). Youssef et al. (16) found that the lipidomics of PE fetuses was altered, manifested as increased triglycerides, cholesterol, and lipoprotein; however, the mechanism by which maternal PE affects neonatal metabolism is not currently well understood, and the metabolic profile of neonates with preeclampsia remains to be refined.

In this study, we conducted a non-targeted metabolomic analysis of umbilical cord serum of PE and non-PE pregnancies to elucidate the metabolic changes in PE neonates. Moreover, the metabolome of small-for-gestational-age (SGA) infants was compared with that of appropriate-for-gestational-age (AGA) infants in PE neonates.

## Materials and Methods

### Study Population

This case-control study was approved by the Institutional Review Board of the Peking Union Medical College Hospital. All women provided written informed consent. Cord serum samples were collected from women whose pregnancies were complicated by PE (*n* = 29) and those without PE (*n* = 32). All participants were singleton pregnancies that delivered their babies at Peking Union Medical College Hospital, China, between January 2020 and August 2021.

PE was diagnosed according to the criteria published in Practice Bulletin No. 202 of the American College of Obstetricians and Gynecologists in 2020. Women in the PE group had the following characteristics after 20 weeks of gestation: (1) Systolic blood pressure (SBP) ≥ 140 mmHg and/or diastolic blood pressure (DBP) ≥ 90 mmHg on two occasions at least 4 h apart; (2) proteinuria ≥ 300 mg/24 h or severe features (thrombocytopenia, impaired liver function, persistent epigastric pain, renal insufficiency, pulmonary edema, new-onset headache, visual disturbances). Given that neonatal metabolic status is related to gestational age, 10 preterm non-PE neonates were selected for the non-PE group to match the gestational age of the two groups. The cause of preterm birth in these 10 cases was a breech presentation complicated by premature rupture of membranes.

Women with fetal abnormalities, chronic kidney disease, pre-pregnancy diabetes, immune system disease, or other chronic medical diseases were excluded.

### Sample Collection

Cord blood samples were obtained right immediately after delivery and were transferred to the laboratory. Samples were centrifuged at 3,000 rpm for 10 min at 4°C. The supernatant serum was extracted, divided into 100 μL aliquots, and stored at –80°C.

### Metabolites Extraction

The 50 μL samples were transferred to EP tubes. After adding 200 μL of extract solution (acetonitrile:methanol = 1:1, containing isotopically-labeled internal standard mixture), the samples were vortexed for 30 s, sonicated for 10 min in an ice-water bath, and incubated for 1 h at –40°C to precipitate proteins. The samples were centrifuged at 12,000 rpm for 15 min at 4°C. The resulting supernatant was transferred into a fresh glass vial for further analysis. A quality control (QC) sample was prepared by mixing an equal aliquot of the supernatant from all samples.

### UHPLC-QE-MS Analysis

LC-MS/MS analyses were performed using a UHPLC system (Vanquish, Thermo Fisher Scientific) with a UPLC BEH amide column (2.1 mm × 100 mm, 1.7 μm) coupled to a Q Exactive HFX mass spectrometer (Orbitrap MS, Thermo). The mobile phase consisted of 25 mmol/L ammonium acetate and 25 mmol/L ammonia hydroxide in water (pH = 9.75) (A) and acetonitrile (B). The auto-sampler temperature was 4°C, and the injection volume was 3 μL.

The QE HFX mass spectrometer was used for continuously evaluation of the full-scan MS spectrum. The ESI conditions were set as follows: sheath gas flow rate, 30 Arb; Aux gas flow rate, 25 Arb; capillary temperature, 350°C; full MS resolution as 60,000, MS/MS resolution as 7,500, collision energy, 10/30/60 in NCE mode; spray voltage as 3.6 kV (UHPLC-QE-MS(+), positive) or –3.2 kV (UHPLC-QE-MS(–), negative), respectively.

Raw data were converted to the mzXML format and processed with an in-house program developed using R and based on XCMS for peak detection, extraction, alignment, and integration. The following steps were taken: Filtering, removing, replacing, normalizing the features.

### Statistical Analysis

The metabolomic data were analyzed by univariate analysis (UVA) and multivariate analysis (MVA) on the UHPLC-QE-MS (+) and UHPLC-QE-MS (–) platforms. For MVA, the dataset was imported into SIMCA (Version 14.1, Umetrics, Umea, Sweden), and the principal component analysis (PCA) and orthogonal partial least squares discriminant analysis (OPLS-DA) models were created. The PCA model was used to analyze the overall distribution of each sample, and the OPLS-DA model was used to analyze the differences in metabolomics between the two groups. For the OPLS-DA model, cross-validation was performed to evaluate the model to avoid bias, and a permutation test was performed to evaluate whether the model was overfitting. Based on the OPLS-DA model, variable importance to projection (VIP) values are reported for each variable (17).

Student’s *t*-test and Mann-Whitney *U*-test were applied for UVA to all metabolites, and the false discovery rate (FDR) was performed to adjust the *p*-value (18). Differential metabolites were selected using MVA and UVA: VIP > 1 and *p* < 0.05 (adjusted *p*-value). The metabolites were annotated using the Human Metabolome Database (HMDB)^1^ (19) and online Kyoto Encyclopedia of Genes and Genomes (KEGG)^2^ (20) database. MetaboAnalyst 5.0^3^ (21, 22) was used for metabolomic data analysis, including hierarchical cluster analysis and heat map visualization.

Based on the summarized differential metabolites screened by the positive and negative modes, MetaboAnalyst (version 5.0) was used for comprehensive pathway analysis (including enrichment and topology analyses). The enrichment of metabolites in the pathways and the impact factors were analyzed. To select metabolic pathways with a high correlation with preeclampsia, the screening criteria were *p* < 0.05 or impact > 0.2.

Figures were created using GraphPad (version 8.0.2) and SIMCA (Version 14.1).

## Results

### Baseline Maternal and Neonatal Characteristics

The characteristics of mothers and newborns are shown in Table 1. Between the PE and non-PE groups, there were no significant differences in maternal age, gravidity, parity, the proportion of assisted reproductive pregnancy, and gestational diabetes mellitus (GDM). There was no significant difference in gestational age at delivery, premature birth rate, cesarean section rate, neonatal Apgar score, or incidence of neonatal complications between the two groups. The SBP, DBP, and body mass index (BMI) of the PE group were significantly higher than those of the non-PE group. The birth weight of the PE neonates was significantly lower than that of the non-PE neonates, and the incidence of SGA was also significantly higher in the PE group.

**TABLE 1:** Demographic and clinical characteristics of the mother and baby: PE vs. non-PE groups.

Parameters	PE (*n* = 29)	non-PE (*n* = 32)	*P*-value
**Maternal characteristics**			
Age at delivery, year	33.6 ± 4.3	34.5 ± 5.4	NS
Gravidity	2.3 ± 1.4	2.2 ± 1.2	NS
Parity	1.3 ± 0.6	1.4 ± 0.5	NS
Firstborn	20 (69.0)	18 (56.3)	NS
Assisted reproductive pregnancy, n(%)	4 (13.8)	5 (15.6)	NS
Early onset PE, n(%)	10 (34.5)	–	–
Late onset PE, n(%)	19 (65.5)	–	–
PE with severe features, n(%)	18 (62.1)	–	–
Highest systolic blood pressure, mmHg	166.3 ± 15.1	128.8 ± 10.6	<0.001
Highest diastolic blood pressure, mmHg	104.3 ± 9.3	78.9 ± 11	<0.001
Chronic hypertension, n(%)	11 (37.9)	–	–
GDM class A1, n(%)	6 (20.7)	7 (21.9)	NS
BMI, kg/m^2^(pre-pregnancy)	24 ± 4.2	21.7 ± 3	0.014
BMI, kg/m^2^(at delivery)	28.7 ± 4	26.5 ± 3.6	0.025
Mgso4 treatment, n(%)	16 (55.2)	3 (9.4)	<0.001
Fetal lung maturation treatment, n(%)	7 (24.1)	4 (12.5)	NS
Cesarean section rate, n(%)	26 (89.7)	24 (75)	NS
**Neonatal characteristics**			
GA at birth, weeks	35.6 ± 3.1	36.8 ± 2.6	NS
Preterm birth rate, n(%)	16 (55.2)	10 (31.3)	NS
Neonatal weight, g	2386.7 ± 916.1	2929.8 ± 639	0.009
Birth weight < 2,500 g, n(%)	16 (55.2)	6 (18.8)	0.003
Birth weight < 1,500 g, n(%)	6 (20.7)	1 (3.1)	0.032
Birth weight < 1,000 g, n(%)	2 (6.9)	1 (3.1)	NS
SGA, n(%)	8 (27.6)	0 (0)	0.003
Apgar/1	9.6 ± 1.5	9.8 ± 0.9	NS
Neonatal complications	15 (51.7)	10 (32.3)	NS
NRDS, n(%)	5 (18.5)	1 (3.2)	NS
NEC, n(%)	0 (0)	2 (6.5)	NS
Neonatal hyperbilirubinemia, n(%)	8 (29.6)	7 (22.6)	NS
IVH, n(%)	11 (40.7)	8 (25.8)	NS
Neonatal infection, n(%)	5 (18.5)	2 (6.5)	NS
Anemia, n(%)	7 (25.9)	4 (12.9)	NS
Neonatal asphyxia, n(%)	2 (7.1)	2 (6.5)	NS

*The results are expressed as mean ± SD or numbers (%) of recruited women; GA, gestational age. SGA, small for gestational age. NRDS, neonatal respiratory distress syndrome. NEC, necrotizing enterocolitis. IVH, intraventricular hemorrhage. NS, not significant.*

### Umbilical Cord Serum Metabolomics Analysis in Non-preeclampsia and Preeclampsia Group

**FIGURE 1:**
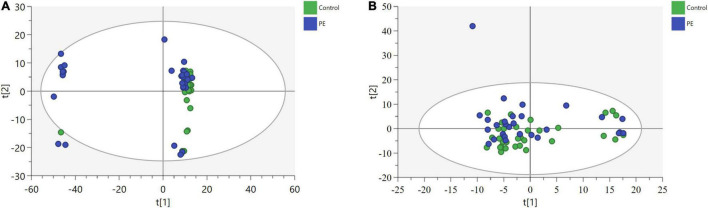
Principle component analysis (PCA) plots show some separation of metabolic profiles between PE (in blue) and non-PE (in green) for (a)UHPLC-QE-MS(+) and (b)UHPLC-QE-MS(–). **(A)** POS [UHPLC-QE-MS(+)], 3 components model:R2X = 0.655, Q2 = 0.537. **(B)** NEG [UHPLC-QE-MS(–)], 9 components model:R2X = 0.503, Q2 = 0.141.

**FIGURE 2:**
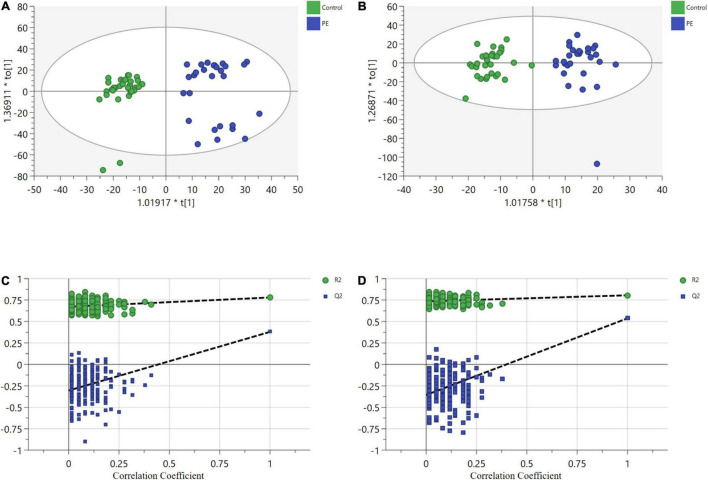
Orthogonal partial least squares discriminant analysis (OPLS-DA) plots show significant separation of metabolic profiles between PE (in blue) and non-PE (in green) for (a1,b1)**. (A)** UHPLC-QE-MS (+) OPLS-DA model, R2X = 0.209, R2Y = 0.78, Q2 = 0.441. **(B)** UHPLC-QE-MS(–) OPLS-DA model, R2X = 0.175, R2Y = 0.805, Q2 = 0.544. **(C)** A permutation test of UHPLC-QE-MS (+) model. The *Y*-axis intercepts were: R2Y (0, 0.6), Q2(0, –0.89). **(D)** A permutation test of UHPLC-QE-MS(–) model. The *Y*-axis intercepts were: R2Y (0, 0.7), Q2 (0, –1.01).

After data processing, 4,698 and 3,621 metabolic characteristics were detected on the positive [UHPLC-QE-MS (+)] and negative [UHPLC-QE-MS(–)] platforms, respectively. PCA of data from the two platforms showed that PE and non-PE neonatal metabolomic profiles were separated to some extent (Figure 1). OPLS-DA analysis on both platforms showed that the metabolomic profile could significantly distinguish PE and non-PE groups (Figure 2). In the positive and negative platforms, the *P*-values of CV-ANOVA were 1.8 × 10–5 and 6.9 × 10–9, respectively, indicating good models. Permutation tests on the two platforms showed no overfitting in the OPLS-DA models. Combined MVA and UVA analysis (VIP > 1 and *p* < 0.05), 1,298 and 838 metabolic characteristics were found to be different in neonatal cord blood on UHPLC-QE-MS (+) and UHPLC-QE-MS (–) platforms, respectively. Among these, 113 and 46 differential metabolites were annotated based on several databases on the UHPLC-QE-MS (+) and UHPLC-QE-MS (–) platforms, respectively.

**FIGURE 3:**
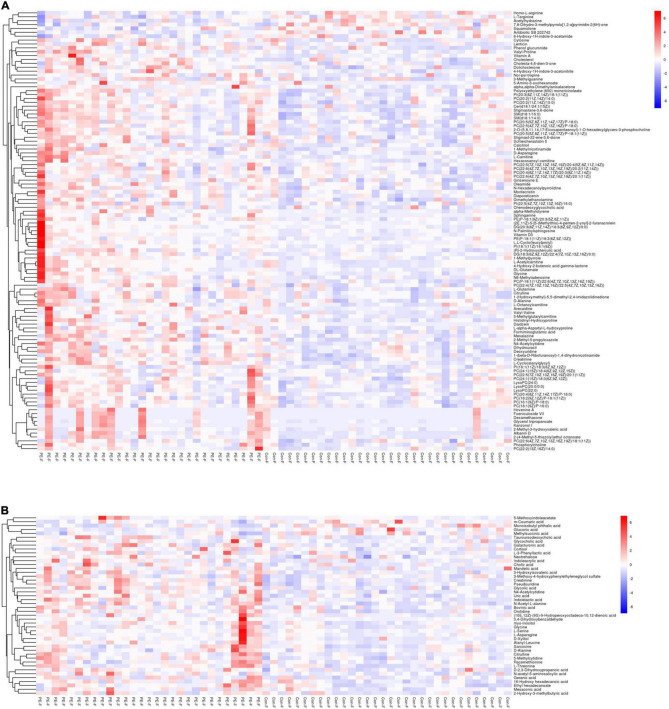
The heatmap showing the hierarchical clustering characteristics of metabolites of PE and non-PE neonates. The color blue indicates decreasing expression, and red indicates increasing expression. The color intensity reflects the corresponding abundance difference. **(A)** Differential metabolite characteristics of the UHPLC-QE-MS (+) model. **(B)** Differential metabolite characteristics of the UHPLC-QE-MS (–) model.

Hierarchical cluster analysis was performed on the screened differential metabolites. The heatmap (Figure 3) showed that differential metabolites could be clustered into two types, enriched in umbilical cord serum of PE and non-PE groups. In the positive platform, 106 metabolites increased in PE (including 59 lipid-related metabolites, 16 amino acid-related metabolites, three nucleotide metabolites, and others), and seven decreased in PE (including three amino acid-related metabolites and others). Among these, creatinine, N4-acetylcytidine, and sphingomyelin (D18:1/16:0) showed significant differences and were enriched in the PE group. In the negative platform, 42 metabolites increased (including 13 amino acid-related metabolites, seven amino acid-related metabolites, four nucleotide metabolites, and others), and four metabolites decreased (including one lipid metabolite, one carbohydrate metabolite, and others). Among these, pseudouridine, uric acid, and indolelactic acid showed significant differences and were enriched in the PE group. Important differential metabolites are listed in Table 2.

**TABLE 2:** Metabolites observed to be significantly different between PE and non-PE groups.

Metabolites	VIP	*P*-value	FC(PE/non-PE)	Platform
N4-Acetylcytidine	2.35	0.0039	1.47	UHPLC-QE-MS(+)
Creatinine	2.20	0.0003	1.41	UHPLC-QE-MS(+)
SM(d18:1/16:0)	2.13	0.0046	1.21	UHPLC-QE-MS(+)
Pseudouridine	2.95	0.0001	1.29	UHPLC-QE-MS(–)
Indolelactic acid	2.71	0.0022	1.75	UHPLC-QE-MS(–)
Uric acid	2.49	0.0006	1.43	UHPLC-QE-MS(–)

*FC, Fold change.*

### Pathway Analysis of Preeclampsia Neonates

**FIGURE 4:**
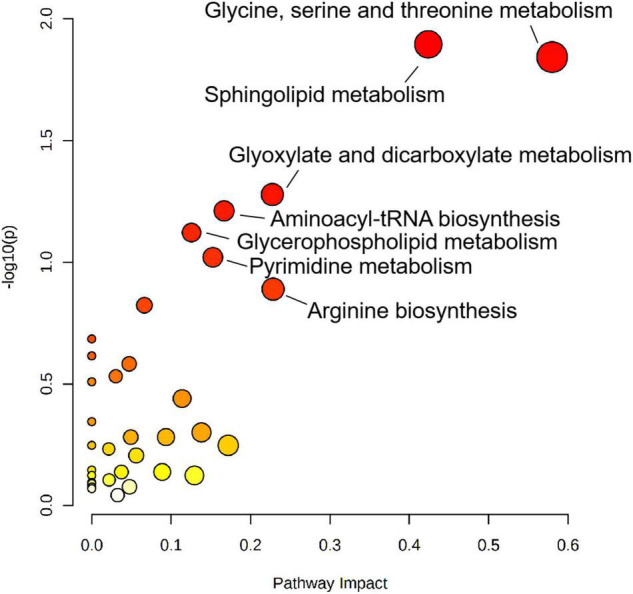
Metabolic pathway analysis results related to differential metabolites between PE and non-PE neonates, presented by bubble plots. Each bubble represents a metabolic pathway. The *x*-axis of the bubble and its size represent the impact factors of the path in topology analysis. The *y*-axis of the bubble and its color represent the *p*-value of enrichment analysis [represented by the negative natural logarithm, -ln(P)]. The color intensity reflects the corresponding *p*-value and the enrichment degree.

**FIGURE 5:**
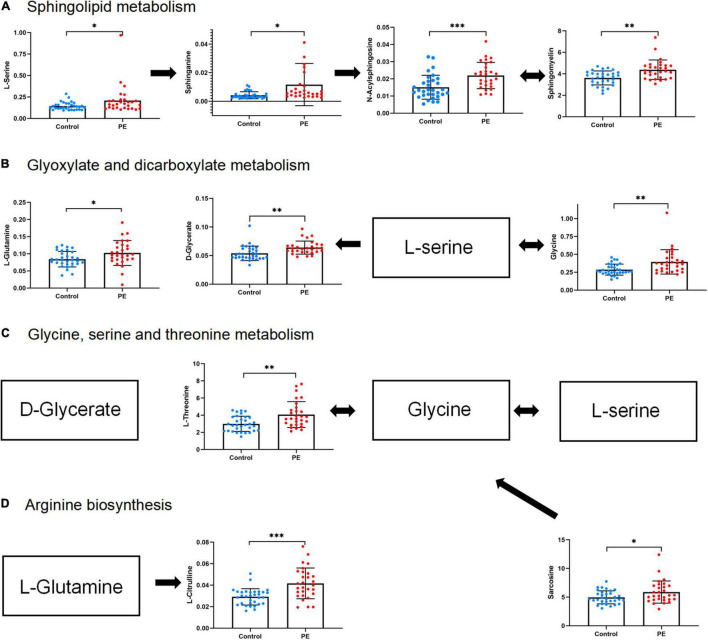
Abundance of differential metabolites in four metabolic pathways related to PE neonates. **(A)** Sphingolipid metabolism. **(B)** Glyoxylate and dicarboxylate metabolism. **(C)** Glycine, serine, and threonine metabolism. **(D)** Arginine biosynthesis. Mean standard deviation and *p*-values are shown in the graph. *represents *p* < 0.05, ^**^represents *p* < 0.01, ^***^represents *p* < 0.001.

To explore the biological functions of differential metabolites, 159 differential metabolites from both UHPLC-QE-MS (+) and UHPLC-QE-MS (–) platforms were integrated into pathway analysis. The 10 pathways with the highest correlation are listed in Table 3. The topological impact factors and enrichment analysis *p*-values of the corresponding pathways are shown in the bubble graph (Figure 4). Screening by the criteria: *p* < 0.05 or impact > 0.2, four metabolic pathways with high influence on PE were selected: glycine, serine, and threonine metabolism, sphingolipid metabolism, glyoxylate and dicarboxylate metabolism, and arginine biosynthesis. The abundance of 10 metabolites in the four pathways above increased in the PE group (Figure 5).

**TABLE 3:** Metabolic pathways related to differential metabolites between PE and non-PE neonates.

Pathways	Hits	*P*-value	-ln(p)	Impact
Glycine, serine, and threonine metabolism	5	0.014	1.84	0.58
Sphingolipid metabolism	4	0.013	1.89	0.42
Arginine biosynthesis	2	0.129	0.89	0.23
Glyoxylate and dicarboxylate metabolism	4	0.053	1.28	0.23
Pentose and glucuronate interconversions	1	0.567	0.25	0.17
Aminoacyl-tRNA biosynthesis	5	0.062	1.21	0.17
Pyrimidine metabolism	4	0.095	1.02	0.15
Nicotinate and nicotinamide metabolism	1	0.502	0.3	0.14
Inositol phosphate metabolism	1	0.753	0.12	0.13
Glycerophospholipid metabolism	4	0.076	1.12	0.13

### Metabolomics Characteristics of Small-for-Gestational-Age in Preeclampsia Neonates

None of the newborns in the non-PE group developed SGA, and the incidence of SGA in the PE group was 27.6%. The PE group was further divided into two subgroups: appropriate for gestational age (AGA) subgroup of PE neonates (PE-AGA group, *n* = 21) and SGA subgroup of PE neonates (PE-SGA group, *n* = 8).

**FIGURE 6:**
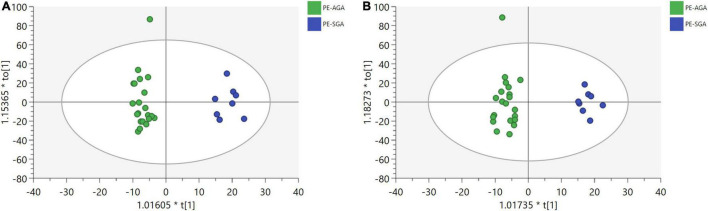
OPLS-DA plots show a significant separation of metabolic profiles between PE-SGA (in blue) and PE-AGA (in green) for (a,b). **(A)** UHPLC-QE-MS(+) OPLS-DA model, R2X = 0.169, R2Y = 0.894. **(B)** UHPLC-QE-MS(–) OPLS-DA model, R2X = 0.199, R2Y = 0.869.

**FIGURE 7:**
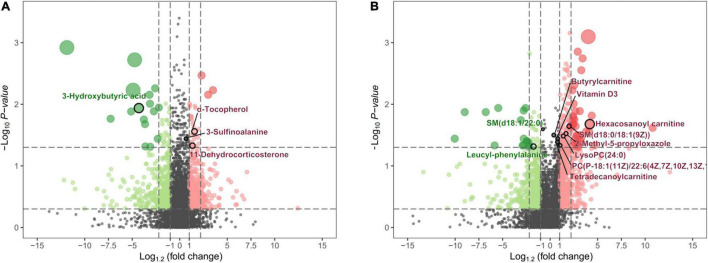
The volcano map shows the *p*-values, fold changes, and VIP-values of all metabolic characteristics. The color green indicates decreasing expression, and red indicates increasing expression. The spot size reflects the corresponding VIP-value. The major metabolites are highlighted in the maps. **(A)** Metabolite characteristics of the UHPLC-QE-MS (+) model. **(B)** Metabolite characteristics of the UHPLC-QE-MS (–) model.

MVA showed that metabolomics could clearly distinguish PE-SGA newborns from AGA newborns on positive and negative ion platforms, as shown in Figure 6. Therefore, UVA and MVA were used to screen for metabolites, and the criteria were as follows: VIP > 1 and *p* < 0.05. Eighteen and seven differential metabolites were found on the UHPLC-QE-MS (+) and UHPLC-QE-MS(–) platforms, respectively (Figure 7).

In the UHPLC-QE-MS (+) platform, 14 metabolites increased in PE-SGA (including eight lipid-related metabolites, two amino acid-related metabolites, and others), and four metabolites decreased in PE-SGA (including two lipid-related metabolites, one amino acid-related metabolite, and others). Hexacosanoyl carnitine was the major significant difference. In the UHPLC-QE-MS (–) platform, three metabolites increased in PE-SGA (including two lipid-related metabolites and one amino acid-related metabolite), and four metabolites decreased in PE-SGA (including one lipid-related metabolite, one amino acid-related metabolite, and one carbohydrate metabolite). 3-hydroxybutyric acid and 3-sulfinoalanine were the most significantly different metabolites.

### Metabolic Pathway Analysis of Small-for-Gestational-Age in Preeclampsia Neonates

**FIGURE 8:**
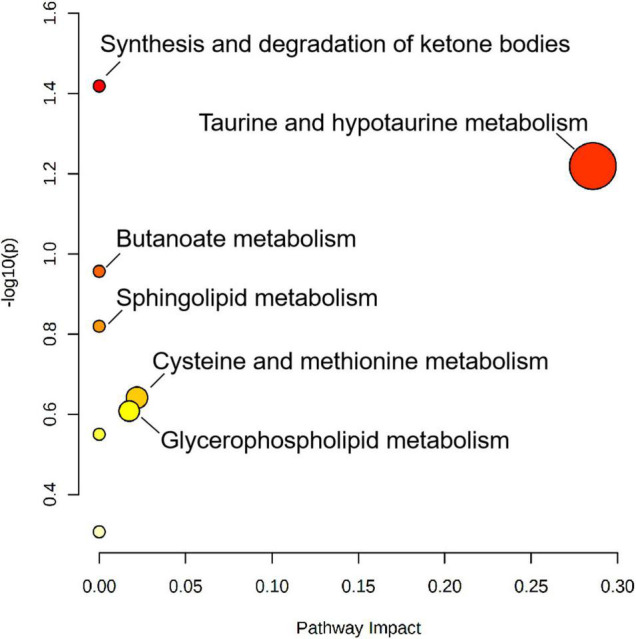
Metabolic pathway analysis results of SGA newborns, presented by bubble graph. Each bubble represents a metabolic pathway. The *x*-axis of the bubble and its size represent the impact factors of the pathway in the topology analysis. The *y*-axis of the bubble and its color represent the *p*-value of enrichment analysis [represented by the negative natural logarithm, -ln(P)]. The color intensity reflects the corresponding *p*-value and the enrichment degree.

**FIGURE 9:**
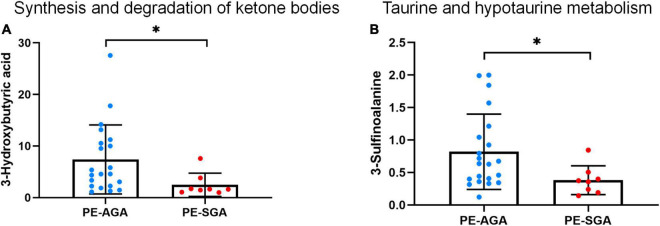
The decreased abundance of differential metabolites in two important metabolic pathways related to PE-SGA neonates. **(A)** Synthesis and degradation of ketone bodies. **(B)** Taurine and hypotaurine metabolism. Mean, standard deviation, and *p*-values are shown in the graph. *represents *p* < 0.05.

A total of 25 different metabolites from the positive and negative platforms were integrated. Six metabolites related to SGA were distributed in eight metabolic pathways, analyzed by MetaboAnalyst 5.0 (specific results are shown in Figure 8). Topological analysis showed that taurine and hypotaurine metabolism had the greatest influence. The metabolic pathways with the highest enrichment degree were those of synthesis and degradation of ketone bodies. The abundance of 3-hydroxybutyric acid and 3-sulfinoalanine, involved in the two pathways as mentioned above, was reduced in SGA neonates (Figure 9).

## Discussion

Preeclampsia is a complex pregnancy disorder that causes damage to fetal growth and development. PE neonates have an average of 5% lower birth weight as compared to children born after an uncomplicated pregnancy, and PE is a significant contributor to preterm birth (23). Preterm birth is the world’s leading cause of neonatal morbidity and mortality. As for short term outcomes, PE is associated with higher rates of infant respiratory distress syndrome, intraventricular hemorrhage, sepsis, bronchopulmonary dysplasia, and neurodevelopmental disability in childhood (24). For long term outcomes, due to abnormal placentation, there is elevated cardiovascular risk in offspring after intrauterine exposure of preeclampsia. Most studies found in offspring exposed to intrauterine preeclampsia, at the age of 9–17 years old, higher systolic blood pressure, other found higher diastolic blood pressure and some both (25). Therefore, it is of great significance to explore the metabolic profiles of neonates with PE and SGA neonates. Based on the UHPLC-QE-MS platform, our study observed metabolic alterations in cord serum involved in the metabolism of amino acids, lipids, and nucleosides in neonates with PE.

Firstly we investigated the metabolites associated with PE newborns, including 6 metabolites that have highest VIP value. We found out that creatinine and uric acid were significantly higher in PE newborns which is consistent with previously reported (14). Moreover, these metabolites also have long been considered to be significantly higher in pregnant women with PE (11). As mentioned before, it is bothering that cord blood might reflect on maternal values and not only the newborns. Creatinine and uric acid were generally considered to be associated with kidney function. It was reported that neonatal creatinine could reflects the level of maternal renal function (26), and predict the onset of neonatal acute kidney injury to some extent (27). Uric acid have effects on fetal growth and kidney development, and lead to a tendency of low-birth-weight infants (28). High levels of uric acid can lead to hypertension-like pathological changes such as arteriole thickening, arteriosclerosis and vasoconstriction in rats, reflecting the correlation between uric acid and hypertension (29). The possible molecular mechanism might due to uric acid can hinder vascular endothelial cell proliferation and function (30). Therefore, the offspring of PE tend to have an increased risk of hypertension in the long term. It may be partly related to the vascular changes resulting from exposure to high levels creatinine and uric acid in fetal and neonatal periods. However, the mechanism of long-term impact in adulthood is unclear.

N4-acetylcytidine and pseudouridine also increased in our PE newborns. Previous studies have found that these metabolites were elevated in the blood of patients with chronic kidney disease, uremia, and pediatric acute kidney injury, respectively (31, 32). These metabolites, cleared by the kidney (33), are related to the decrease of glomerular filtration rate (34) and may reflect the renal function of patients to some extent.

Indoleolactic acid is a tryptophan metabolite of an essential amino acid during pregnancy that meets the needs of fetal growth and development (35). Consistent with our study, Jääskeläinen et al. also found that indoleolactic acid increased in umbilical cord blood of PE newborns (14). In addition, Morita et al. found that PE can lead to an elevated concentration of tryptophan metabolites levels, including indoleolactic acid, in neonatal serum (15). Previous studies have shown that phenyllactate dehydrogenase and acyl-CoA dehydrogenase in the placenta can convert indoleolactic acid to indolepropionic acid and that PE may inhibit the activity of both enzymes, leading to the accumulation of indoleolactic acid (36). In addition, indoleolactic acid has been found to help maintain cell stability in human umbilical vein endothelial cells, antagonizing the abnormal endothelial function of PE. Besides, indoleolactic acid show an anti-inflammatory effect in the intestine of premature infants, and its low level may be implicated in irritable bowel syndrome and NEC (37). Therefore, increased indoleolactic acid seems to be a self-protective factor for newborns with PE.

Further, we use metabolites to conduct pathway analysis and PE newborns were associated with the following pathways.

(1) ***Glycine, serine, and threonine metabolism.*** Glycine is located at the central position in the glycine, serine, and threonine pathways. It can also be interconverted with l-serine and l-threonine. Sarcosine is metabolized to glycine by sarcosine dehydrogenase. A latest study of neonates also found elevated glycine in SGA neonates (38). From the perspective of nutrition, it is speculated that this amino acid alteration, we found, may reflect the enhanced decomposition of protein to make up for the insufficient energy supply of PE placenta. Glycine may alleviate inflammatory injury induced by ischemia and reperfusion and inhibit interleukin production (39, 40). It was found that glycine in FGR newborns was relatively increased, and glycine concentration in umbilical cord blood was inversely proportional to oxygen content (41). The change in glycine levels in cord blood may reflect the adaptation of newborns with PE in the ischemia and hypoxia state. It is generally believed that abnormal vasculogenesis in the PE placenta may lead to relative ischemia and hypoxia in the fetal supply. This is the first study to determine the characteristics of the glycine metabolic pathway in PE newborns.

(2) ***Arginine biosynthesis.*** Our data showed that arginine biosynthesis was altered in PE neonates due to L-citrulline and L-glutamine’s increased abundance. Glutamine is a precursor of citrulline, synthesized in the gut. Citrulline is mainly converted to arginine in the kidneys in adults (42). In preterm neonates, citrulline maybe directly converted in the guts due to imperfect gut-kidney organ circulation (43). A previous study found that citrulline was slightly higher in PE cord blood than in non-PE cord blood (44). Some studies have shown that citrulline antagonizes hypertension and reduces arterial tension by activating the citrulline-nitric oxide cycle (45). However, a recent systematic review showed no strong evidence of citrulline in fighting hypertension, and more clinical studies are needed (46). In addition, glutamine is a potential marker of brain injury. In previous studies, among newborns with hypoxic-ischemic brains, those with poor outcomes had higher glutamine concentrations (47). Glutamine is also associated with severe brain edema (48). Therefore, glutamine may help in the early recognition and treatment of neonatal PE brain injury.

(3) ***Sphingolipid metabolism.*** N-Acylsphingosine, a ceramide, is a sphingolipid with antiangiogenic properties. Ceramide, sphingomyelin, and sphinganine play important roles in the pathogenesis of cardiovascular diseases and regulation of endothelial cell function (49). A study in newborns found an increase in ceramide in feces before the onset of NEC, and abnormal sphingolipid metabolism may change the permeability of intestinal cells, leading to inflammatory diseases (50). Ceramide also promotes apoptosis (51). The ceramide concentration is often higher in patients with Alzheimer’s disease, leading to a wide range of neuronal apoptosis and cognitive impairments (52). However, the role of ceramide in neonatal cognition and brain development remains unclear.

PE tend to have a high incidence of low birth weight and SGA (53). Our study also reflected this feature. As mentioned above, uric acid is an important risk factor for low birth weight infants (28). The free activity of uric acid in maternal-fetal circulation can hinder the proliferation and function of endothelial cells, causing interference in the fetal growth and organ development (30, 54). Another neonatal metabolomics study has found a correlation between aberrant sphingolipids and SGA (55). Growth restriction is associated with placental cell apoptosis, which may be regulated by sphingolipids (56, 57). The abnormal sphingolipid metabolism, observed in our study, may increase the signaling of apoptosis, limiting the fetal growth. On the other hand, it is commonly accepted that the main pathophysiological mechanism of SGA is the reduced uterine-placenta blood perfusion. Poor formation of spiral arteries, associated with PE, can also lead to the similar damage, increasing the incidence of fetal growth restriction (58).

In order to explore the potential molecular mechanism underling PE associated adverse neonatal outcomes, we investigated the metabolites associated with SGA neonates. We observed abnormalities in amino acid, lipid, and ketone body metabolisms in SGA neonates. Carnitine is a carrier of lipid transport, and lipids move from the cytoplasm to the mitochondria, where they are metabolized. Mitochondrial dysfunction makes it difficult to consume fat, resulting in carnitine accumulation (59). Beken et al. also found abnormal carnitine in SGA neonates, including reduced propionyl carnitine and methylglutaryl carnitine (38). Another study reported an increased carnitine concentration in neonates with a high body fat rate (60). Combining our study with other studies, we speculate that SGA neonates have abnormal lipid metabolism and fat accumulation. Individuals born SGA are at high risk of long-term obesity (61). Although long-term growth is affected by many factors, such as the environment and diet, metabolic abnormalities originating from newborns may be one of the reasons.

Previous studies have indicated that fetuses exhibit growing energy requirements during fetal growth in normal pregnancy, including increased glucose and fatty acids consumption. Fetal ketogenesis is a response to the potential risk of hypoglycemia (62) and is also an important backup supplement for fetal energy supply, resulting in the accumulation of acetoacetic and hydroxybutyric acids (63). A positive correlation between birth weight and hydroxybutyric acid concentration in umbilical cord blood was also observed in studies by Mansel et al. (64) and Lowe et al. (65). In this study, the low level of 3-hydroxybutyric acid indicates that the metabolic pathway of ketone bodies in SGA neonates was impaired. Inadequate energy supply and placental nutritional disorders can result in adaptive changes in newborns that manifest as weight loss.

In the taurine metabolic pathway, 3-sulfinoalanine produces taurine. Taurine metabolism plays an important role in facilitating substance exchange and nutrient supply in the placenta. One study found a positive association between maternal taurine intake and neonatal height in pregnancy (66). Previous studies have shown that low taurine levels can limit neonatal neurodevelopment, retinal development, and intestinal absorption function (67). Animal experiments have shown that taurine supplementation may have a protective effect on neural development in neonatal rats (68). However, little research has been conducted on human neonates due to limitations of ethical requirements. The latest meta-analysis of taurine supplementation did not confirm its role and showed no significant effects on the growth and development of newborns (69). On the other hand, taurine is also an important biomarker of individual neurodegeneration (70). Due to its antioxidant property, high levels of taurine can reduce cognitive impairment caused by neurodegeneration lesions (71, 72). It is observed, children with SGA have a relatively elevated risk of cognitive impairment, learning difficulties, and even cerebral palsy in the long term (73). Low abundance of taurine in our study may partially explain this problem. Therefore, detection of taurine level may be beneficial for timely evaluation and prevention of neurological diseases, not only for newborns, but also for offspring.

The results of our study reflect the value of metabolomics in evaluating the pathological mechanisms of neonates with PE. These metabolites have diverse functions. Not only can a single metabolite reflect the inherent characteristics of the disease, but a variety of related metabolites can also better reflect the changes in a certain biochemical mechanism. These findings are insufficient to fully reflect all the changes involved in the pathogenesis of PE neonates; however, they still provide a basis and perspective for further research on PE neonates. This is the first metabolomic study of SGA neonates with PE, although it was not the primary objective of our study. This study recorded and statistically analyzed metabolic characteristics and recent complications of PE neonates. However, the long-term effects of metabolic characteristics on neonates remain unclear. Further studies on the risk of related metabolic or cardiovascular diseases based on metabolomic profiles may be needed in the future.

## Data Availability Statement

The original contributions presented in the study are included in the article/Supplementary Material, further inquiries can be directed to the corresponding author.

## Ethics Statement

The studies involving human participants were reviewed and approved by the Institutional Review Board of Peking Union Medical College Hospital. Written informed consent to participate in this study was provided by the participants’ legal guardian/next of kin.

## Author Contributions

XW, JL, and YS conceived and designed the study. XW and JL collected the serum materials and wrote the manuscript. JL and XH performed the experiments. XW, JL, and XH analyzed and modified the data. YS provided advice, assistance, review and editing. All authors read and approved the manuscript.

## Conflict of Interest

The authors declare that the research was conducted in the absence of any commercial or financial relationships that could be construed as a potential conflict of interest.

## Publisher’s Note

All claims expressed in this article are solely those of the authors and do not necessarily represent those of their affiliated organizations, or those of the publisher, the editors and the reviewers. Any product that may be evaluated in this article, or claim that may be made by its manufacturer, is not guaranteed or endorsed by the publisher.

## Footnotes

1
https://hmdb.ca/

2
https://www.kegg.jp/

3
https://www.metaboanalyst.ca/
